# 1664. The Impact of Levofloxacin Prophylaxis on Empiric Intravenous Antibiotic Use in Pediatric Hematopoietic Stem Cell Transplant Recipients

**DOI:** 10.1093/ofid/ofad500.1497

**Published:** 2023-11-27

**Authors:** Michael Prodanuk, Kathryn E Timberlake, Alicia Koo, Yogi Chopra, Donna Wall, Michelle Science

**Affiliations:** Hospital for Sick Children, University of Toronto, Toronto, Ontario, Canada; The Hospital for Sick Children, Toronto, ON, Canada; The Hospital for Sick Children, Toronto, ON, Canada; The Hospital for Sick Children, Toronto, ON, Canada; The Hospital for Sick Children, Toronto, ON, Canada; The Hospital for Sick Children, Toronto, ON, Canada

## Abstract

**Background:**

Clinical trials have demonstrated that levofloxacin prophylaxis during periods of neutropenia in hematopoietic stem cell transplantation (HSCT) reduces the frequency of bacteremia in adults and febrile episodes in children. Therefore, levofloxacin prophylaxis may also have a role in reducing empiric intravenous antibiotic (EIA) use.

**Methods:**

This retrospective review assessed the impact of levofloxacin prophylaxis for patients < 18 years undergoing HSCT at a Canadian children’s hospital. The primary outcome was antibiotic days of therapy (DOT) during the pre-engraftment period comparing the pre-levofloxacin era (Jan 1, 2019–Jun 30, 2020) to the levofloxacin era (Jul 1, 2020–Dec 31, 2021). Patients were excluded if they were receiving EIA at the time of transplant, received chimeric antigen receptor T-cell therapy, or if levofloxacin use was discordant with their era. Secondary outcomes included the number of positive blood cultures and clinical deterioration episodes (clinical change resulting in blood culture draw and initiation/change of EIA).

**Results:**

Fifty-four of 152 patients (36.5%) and 55 of 147 patients (37.4%) were included in the pre-levofloxacin and levofloxacin eras respectively. The most common reasons for exclusion were EIA use at the time of transplant and levofloxacin use discordant with the patient's era (Figure 1). Baseline characteristics were not significantly different between groups (Table 1). Mean DOT/pre-engraftment days (%) were significantly lower in the levofloxacin era for piperacillin-tazobactam (53.3 vs. 38.3, p=0.004) and amikacin (1.7 vs. 0.1, p=0.03), while there was no significant difference for meropenem, vancomycin, or all other antibiotics combined. There was also no significant difference in the number of positive blood cultures (11 vs. 7, p=0.35) or clinical deterioration episodes (55 vs. 66, p=0.1).

**Figure:**
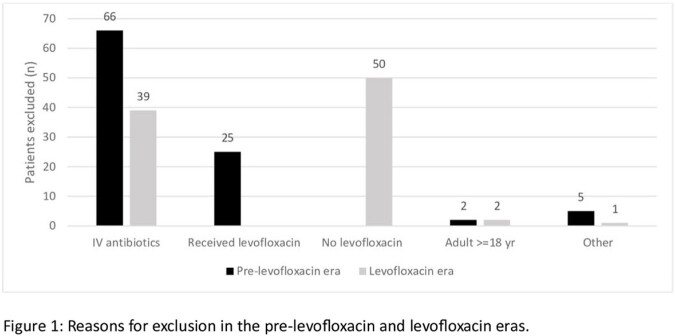

**Figure:**
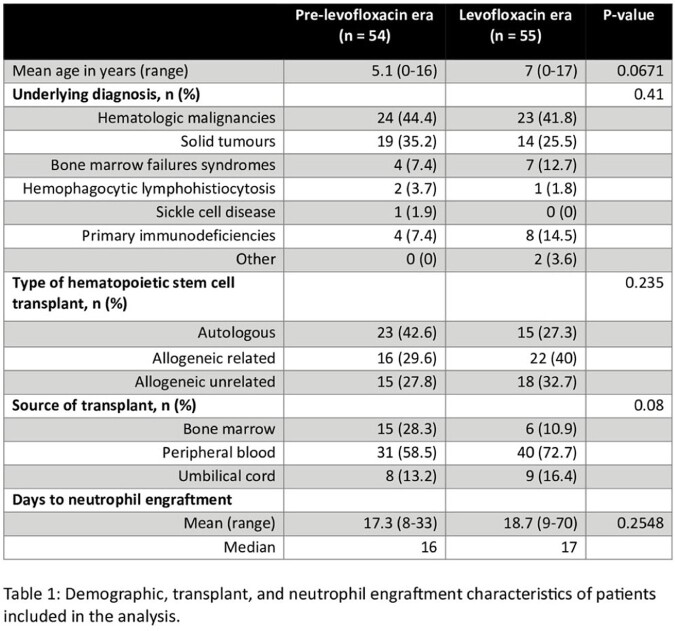

**Conclusion:**

Levofloxacin prophylaxis in children undergoing HSCT reduced percent pre-engraftment days on piperacillin-tazobactam and amikacin, with no significant impact on the number of positive blood cultures or clinical deterioration episodes. Therefore, levofloxacin prophylaxis may facilitate antimicrobial stewardship activities through reduced use of certain broad-spectrum antibiotics.

**Disclosures:**

**Kathryn E. Timberlake, PharmD**, Avir Pharma: Advisor/Consultant

